# Effectiveness of the Healthy Lifestyles Programme (HeLP) to prevent obesity in UK primary-school children: a cluster randomised controlled trial

**DOI:** 10.1016/S2352-4642(17)30151-7

**Published:** 2018-01

**Authors:** Jenny Lloyd, Siobhan Creanor, Stuart Logan, Colin Green, Sarah G Dean, Melvyn Hillsdon, Charles Abraham, Richard Tomlinson, Virginia Pearson, Rod S Taylor, Emma Ryan, Lisa Price, Adam Streeter, Katrina Wyatt

**Affiliations:** a Institute of Health Research, University of Exeter Medical School, University of Exeter, Exeter, UK; b Sport and Health Sciences, University of Exeter, Exeter, UK; c Peninsula Clinical Trials Unit and Medical Statistics, Plymouth University Peninsula Schools of Medicine and Dentistry, Plymouth, UK; d Royal Devon and Exeter NHS Foundation Trust, Exeter, UK; e Public Health Devon, Devon County Council Commissioning Headquarters, Exeter, UK; f ISCA Academy, Earl Richards Road South, Exeter, UK

## Abstract

**Background:**

Although childhood overweight and obesity prevalence has increased substantially worldwide in the past three decades, scarce evidence exists for effective preventive strategies. We aimed to establish whether a school-based intervention for children aged 9–10 years would prevent excessive weight gain after 24 months.

**Methods:**

This pragmatic cluster randomised controlled trial of the Healthy Lifestyles Programme (HeLP), a school-based obesity prevention intervention, was done in 32 schools in southwest England. All state-run primary and junior schools in Devon and Plymouth (UK) with enough pupils for at least one year-5 class were eligible. Schools were assigned (1:1) using a computer-generated sequence to either intervention or control, stratified by the number of year-5 classes (one *vs* more than one) and the proportion of children eligible for free school meals (<19% [the national average] *vs* ≥19%). HeLP was delivered to year-5 children (ages 9–10 years) over 1 year, and included dynamic and interactive activities such as physical activity workshops, education sessions delivered by teachers with short homework tasks, drama sessions, and setting goals to modify behaviour (with parental support and one-to-one discussions with HeLP coordinators). The primary outcome was change in body-mass index (BMI) standard deviation score (SDS) between baseline and 24 months, analysed in children with BMI data available for both timepoints. This study is registered with the International Standard Randomised Controlled Trial register, number ISRCTN15811706, and the trial status is complete.

**Findings:**

Between March 21, 2012, and Sept 30, 2013, 32 eligible schools with 1324 children were recruited, of which 16 schools (676 children) were randomly assigned to the HeLP intervention and 16 schools (648 children) to control. All schools that began the trial completed the intervention, and 1244 children (628 in intervention group and 616 in control group) had BMI data at both baseline and 24 months for the primary outcome analysis. Mean BMI SDS was 0·32 (SD 1·16) at baseline and 0·35 (1·25) at 24 months in the intervention group, and 0·18 (1·14) at baseline and 0·22 (1·22) at 24 months in the control group. With adjustment for school-level clustering, baseline BMI scores, sex, cohort, and number of year-5 classes and socioeconomic status of each school, the mean difference in BMI SDS score (intervention–control) at 24 months was −0·02 (95% CI −0·09 to 0·05), p=0·57. One parent reported an adverse event related to their child's eating and activity behaviours, but agreed for the child to continue trial participation after discussion with the chief investigator.

**Interpretation:**

Despite a theoretically informed and extensively piloted intervention that achieved high levels of engagement, follow-up, and fidelity of delivery, we found no effect of the intervention on preventing overweight or obesity. Although schools are an ideal setting in which to deliver population-based interventions, school-based interventions might not be sufficiently intense to affect both the school and the family environment, and hence the weight status of children. Future research should focus on more upstream determinants of obesity and use whole-systems approaches.

**Funding:**

UK National Institute for Health Research, Public Health Research Programme.

## Introduction

Childhood obesity is considered by WHO to be one of the most serious public health challenges worldwide for the 21st century,^1^ and research has therefore largely focused on preventive approaches. The UK Government^2^ views schools as central to tackling the obesity crisis because they are an ideal setting in which to actively engage children and their families across the socioeconomic spectrum to improve diet and physical activity behaviours. In 2017, the Government has pledged to invest the revenue from the sugar levy (a tax on sugar-sweetened beverages) into school-based programmes to encourage physical activity and balanced diets.^3^

However, findings from systematic reviews^4^, ^5^ showed that the effectiveness of school-based obesity prevention programmes is inconclusive and contradictory: interventions are highly heterogeneous in design and most studies have methodological weaknesses, such as insufficient statistical power, high levels of attrition, differential uptake and follow-up, and only short-term follow-up (12 months on average).^4^, ^5^, ^6^

**Research in context**

**Evidence before this study**

Before the Healthy Lifestyles Programme (HeLP) was designed, a 2005 Cochrane systematic review recommended that interventions to prevent childhood obesity should have a rigorous assessment design that enables sufficiently powered analysis of what is working or not and for whom the intervention is working, and that stakeholders should be included in the development of the programme. A 2009 Cochrane systematic review of school-based obesity prevention interventions was unable to make definitive conclusions because studies were heterogeneous and only five of 38 studies followed up participants for more than 12 months. In 2011, a meta-analysis of 27 studies aiming to prevent obesity in children aged 6–12 years found some evidence for the effectiveness of combined diet and physical activity interventions; policies and strategies that appeared to be promising included providing support for teachers to implement health promotion strategies and activities in schools, and parental support that encourages healthy behaviour in children. In 2015, a review of childhood obesity prevention studies showed a moderate strength of evidence to support the effectiveness of school-based interventions. The Active for Life Year 5 (AfLY5) cluster randomised controlled trial tested a school-based intervention for children aged 9–10 years. The programme included lessons and child–parent interactive homework plans and was adapted from the American Planet Health Programme. No effect on weight status or on objectively measured physical activity or diet was found at 12 months.

**Added value of this study**

HeLP was developed using an intervention mapping approach involving relevant behaviour change theories, best available evidence, and extensive involvement of teachers, head teachers, families, and children. To our knowledge, our study is the most comprehensive obesity prevention trial to date, involving a large, nationally representative sample of children aged 9–10 years and using prespecified standard methods for randomisation and analysis. The HeLP intervention was delivered with a high degree of fidelity and engaged more than 90% of children and 75% of their families. The evidence from this study therefore has internal validity and is potentially widely applicable.

**Implications of all the available evidence**

Our results highlight the tension facing childhood obesity prevention programmes, because schools are an ideal setting in which to deliver population-based interventions. However, taking into account the inconclusive evidence from the most recent systematic reviews and the results from both ours and the AfLY5 trial, we believe that individually focused school-based interventions targeting a single age group are unlikely to be sufficiently intense or family focused to affect the weight status of children. Future research should focus on more upstream determinants of obesity and use whole-systems approaches.

In line with WHO's Health Promoting Schools framework,^7^ we developed the Healthy Lifestyles Programme (HeLP), consisting of activities that were compatible with the English national school curriculum and promoting messages in a manner that could affect both the wider school culture and specific behaviours of children and their families. The objective of this trial was to ascertain whether HeLP was effective in preventing childhood obesity.

## Methods

### Study design

This two-arm, pragmatic, school-based, cluster randomised controlled trial with masked outcome assessment was done in 32 schools in the southwest of England. Ethics approval was given by the Peninsula College of Medicine and Dentistry Research Ethics Committee (reference number 12/03/140), and research governance approval by the Royal Devon and Exeter National Health Service Trust (study number 1304762). The full trial protocol has been published^8^ and is also available online.

### Participants

All state-run primary and junior schools in Devon and Plymouth (UK) with enough pupils for at least one year-5 class (children aged 9–10 years) were eligible. Schools for children whose additional needs cannot be met in a mainstream setting were excluded because they were unlikely to be teaching the standard national curriculum, around which the intervention had been designed. Schools willing to participate and fulfilling the inclusion criteria were then purposefully sampled by JL and KW to represent a range of school sizes (one to three year-5 classes), locations (urban and rural), and socioeconomic status (<19% and ≥19% of children eligible for free school meals). We aimed to have half of the schools in the trial with at least the national average proportion of pupils eligible for free schools meals (19% at the time of recruitment of schools). Before randomisation, head teachers from all schools gave written informed consent.

To accommodate the logistics and personnel required for delivering the week-long drama component of the intervention to each year-5 class, the trial ran across two cohorts (cohort 1 commenced the trial in September, 2012, and cohort 2 in September, 2013). Schools that were eligible but not sampled for the trial were asked if they were prepared to go on a waiting list, in case any of the schools allocated to participate in cohort 2 dropped out during the interim 1-year period before commencing participation.

All children in all year-5 classes within each recruited school were invited to participate, and their parents or carers could choose to opt their child out before baseline measurements were taken (full details in protocol).^8^ All children who were on the registration list at one of the recruited schools at the start of the autumn term 2012 (for cohort 1) or 2013 (for cohort 2), and whose parents or carers did not complete an opt-out form, were classed as participants.

### Randomisation and masking

The trial manager (JL) was responsible for recruiting schools via the Devon Association of Primary School Heads and local primary school learning community meetings. Schools were randomly allocated (1:1) to the intervention or control group using a computer-generated sequence using two school-level stratification factors: one versus more than one year-5 class and the proportion of children eligible for free school meals (<19% *vs* ≥19%). Randomisation was done by a statistician (RST) in the UK Clinical Research Collaboration-registered Peninsula Clinical Trials Unit immediately after all schools had been recruited in 2012, but each school's allocated group (intervention or control) was not communicated to the schools, parents, or researchers until after baseline measurements had been taken. RST ensured that numbers of control and intervention schools were equal in both cohorts to facilitate trial delivery.

Because of the nature of the intervention, school staff, children, and individuals delivering the intervention could not be masked to group allocation. Anthropometric measures at 18 and 24 months were collected by independent, masked, trained assessors who were not involved in the trial. We made a timeline cluster diagram for the trial to show the masking procedures for each measure at each timepoint (appendix). At the 24-month primary endpoint, when secondary schools contained a mixture of children from intervention and control primary schools, an assessment of the fidelity of assessor masking was made to ascertain whether a child had revealed their group allocation during the measurement process. If the child had revealed their group allocation in any way then this was recorded by the assessor.

### Procedures

In schools assigned to the intervention group, HeLP was delivered to year-5 children (ages 9–10 years) over three school terms (roughly 12 weeks per term). HeLP consisted of four phases, which were ordered to enable and support behaviour change by targeting school and family environments and giving children the strategies and motivation to improve their snacking and activity-related behaviours (panel). Findings from the first pilot of the intervention^9^ (delivered to children aged 8–11 years, school years 4–6) showed that year-5 children were most receptive to the healthy lifestyle messages and engaged their parents to the greatest extent. Also, the school could more feasibly run the HeLP activities in year 5 than year 6, when the curriculum focused on standard assessment tasks. As a result, the intervention was targeted at students in year 5, while also trying to affect the wider school environment.^9^ The programme delivered a general healthy lifestyle message with a focus on behaviours such as the consumption of sugar-sweetened beverages, healthy and unhealthy snacking, physical activity, and reducing screen time. An overarching message promoted was the 80/20 rule, which recommended eating healthily and being active at least 80% of the time. HeLP was designed to fit in with the national curriculum at key stage 2, and all lessons and drama sessions included learning objectives relating to personal social and health education, science, numeracy, and literacy (see further details in the appendix). The development, content, and theoretical underpinning of the intervention have been published previously.^10^

Panel **Summary of intervention phases**

**Phase 1: creating a supportive context (spring term of year 5)**

The aim is to establish relationships between all stakeholders (ie, head teachers, teachers, support staff, children, and parents) and raise awareness of the programme. Professional sports people and dancers run practical workshops and introduce the importance of healthy lifestyles to create a buzz in the school and set a positive atmosphere for future activities. At the end of this phase, children showcase the skills they have learnt in a parent assembly, in which parents are given further information about the programme by the Healthy Lifestyles Programme (HeLP) coordinator.

**Phase 2: intensive healthy lifestyle week (summer term of year 5)**

Education lessons are delivered by the class teacher each morning and interactive drama activities by a local drama group every afternoon during the week. Short and simple homework tasks are given at the end of each session for the children to complete in time for the next session. The drama framework is built around four characters (Disorganised Duncan, Football Freddie, Snacky Sam, and Active Amy), each represented by an actor, whose attributes relate to the three key programme behaviours (reducing unhealthy snacking, increasing physical activity, and reducing sedentary activities). Children are asked to choose the character they felt they most resemble and, throughout the week, work closely with that actor to help the character change their behaviour. These sessions are dynamic and interactive and involve role play, games, dance, problem solving, food tasting, and forum theatre. The themes for each lesson and drama session are as follows: energy in and out, overcoming temptation, decision making and responsibility, food marketing, and goal setting.

**Phase 3: personal goal setting with parental support (summer term of year 5)**

Children are encouraged to reflect on their own behaviours and set goals (based on the HeLP messages) with their parents. After reflection with parents, each child has a 10-min one-to-one goal-setting discussion with a HeLP coordinator. A sheet with each child's goals and the name and attributes of the character the child worked with is sent directly home to parents and a copy is also kept at school in the children's healthy lifestyles folder.

**Phase 4: reinforcement activities (autumn term of year 6)**

A range of components were used to refocus the children and their parents on the HeLP messages and behaviour change strategies. This phase includes a further lesson led by the class teacher, a drama workshop delivered by the actors, an assembly delivered by the class to the whole school about the programme, and a second one-to-one goal discussion with a HeLP coordinator.

One year-5 class per school had their physical activity levels assessed using accelerometers. If a school had more than one year-5 class, a computer-generated sequence was used to randomly select one class. Children were asked to wear a waterproof triaxial accelerometer continuously (including at night) for 8 consecutive days on the wrist of their non-dominant arm.

Schools assigned to the control group continued standard education provision throughout their participation in the trial, and had no access to any of the HeLP resources and scripts, which have not been published and were not made available by the research team beyond the intervention schools. Control schools each received £1000 for their participation following the collection of 18-month data.

Baseline assessments were done in the autumn term of school year 5 between October and November (2012 for cohort 1 and 2013 for cohort 2). Delivery of the intervention began the following term (January, 2013, for cohort 1 and January, 2014, for cohort 2). Follow-up outcome measures were taken at 18 and 24 months after baseline. All measurements were taken at school during the school day. Fidelity of intervention delivery was assessed in relation to both content and the quality of delivery (appendix).

### Outcomes

The primary outcome was the change in body-mass index (BMI) standard deviation score (SDS) between baseline and 24-month follow-up. BMI was calculated and converted to centiles using the LMS method for constructing normalised growth standards.^11^ Categorisations of underweight, normal, overweight, or obese were based on the definitions from Cole and colleagues.^12^

Secondary outcomes were BMI SDS at 18 months; the percentage of children classified as underweight, healthy weight, overweight, and obese at 18 and 24 months; waist circumference SDS at 18 and 24 months; percentage body fat SDS at 18 and 24 months; physical activity measured using accelerometry at 18 months; and self-reported scores for the number of different types of energy-dense snacks, healthy snacks, healthy foods (positive food markers), and unhealthy foods (negative food markers) consumed per day using the validated Food Intake Questionnaire (FIQ)^13^ at 18 months (appendix).

Details of methods of data collection for the anthropometric and behavioural measures are in the appendix. An adverse event was considered to include unusual dieting or physical activity behaviours or noticeable weight loss. Any adverse event could be reported by school staff, parents, HeLP coordinators, or actors.

### Statistical analysis

Our sample size calculation assumed a mean of 35 children aged 9–10 years per school, with coefficient of variance of 0·5 and an intraclass correlation coefficient of 0·02. To have 90% power, with a two-sided 5% significance level, to detect a between-group difference in BMI SDS of 0·25 units at 24 months, assuming an SD of 1·3 and adjusting for baseline BMI SDS (assumed within-child correlation of 0·8), we needed to have 24-month outcome data from at least 762 children. Allowing for up to 20% loss to follow-up, we aimed to recruit 28 schools with at least 952 children.

The primary analyses were done in children with BMI data available for both baseline the 24-month follow-up by a statistician masked to the allocated group. Because of the high levels of completeness of data and low proportion of children categorised as non-compliers, the multiple imputation approach for handling missing outcome data was replaced by a best-case and worst-case scenario, and the planned complier average causal effect analyses were dropped.^14^

All comparative analyses allowed for the clustering of children within schools^15^ using a likelihood-based random-effects regression modelling approach that uses all available data and provides valid estimates of the effect of the intervention, when data are assumed to be missing at random. Most of the outcomes were of a continuous nature and thus linear models were fitted. Weight status was analysed using a random-effects ordinal logistic regression model with three categories (underweight or healthy weight, overweight, and obese) and a random-effects logistic regression model with two categories (underweight or healthy weight and overweight or obese); for simplicity, only the results of the logistic regression models are reported here.

All primary comparative analyses were adjusted for the two school-level stratification factors (proportion of children eligible for free school meals and number of year-5 classes), cohort, sex, and the baseline values for the outcome under consideration, when available. Adjusted between-group mean differences (intervention minus control) and odds ratios (intervention *vs* control), with corresponding 95% CIs, were calculated for all outcomes. p values are two sided and were considered significant at 0·05 or less. Between-group differences with adjustment only for clustering are given for completeness.^15^ The intraclass correlation coefficient (with 95% CI) from the random-effects regression models are reported for all outcomes.

Additional preplanned exploratory subgroup analyses were done to assess whether any effect of the HeLP intervention on the primary outcome was modified by sex, baseline BMI SDS, number of year-5 classes within a school, child-level socioeconomic status, or trial entry time (ie, cohort effect). We also fitted a repeated measures model to all the BMI SDS data at baseline, 18 months, and 24 months, including effects of time and the interaction term between allocated group and time, to assess whether the effect of the intervention differed over time.

For the physical activity analysis, children were included if they had data on at least three weekdays and one weekend day, each with a minimum of 10 h per day.^16^ In the analyses, non-wear of the accelerometer was established by at least two accelerometer axes with an SD less than 13 mg and a range less than 50 mg over a 60-min period, using moving increments of 15 mins.^17^ Accelerometers were set to record at 85·7 Hz and data were downloaded using GeneActiv PC software, version 1.4, and analysed using the GGIR software package for *R*.

We also did parallel economic and process analyses (see analysis plan in the protocol), which will be reported separately. A detailed statistical analysis plan has been published.^14^ All analyses were done in Stata, version 14.0, unless otherwise stated.

This trial is registered with International Standard Randomised Controlled Trials, number ISRCTN15811706.

### Role of the funding source

The funders had no role in study design, data collection, data analysis, data interpretation, or writing of the report. JL, KW, and SC had full access to all the data. All authors commented on drafts and approved the final report, and JL had final responsibility for the decision to submit for publication.

## Results

**Figure 1:**
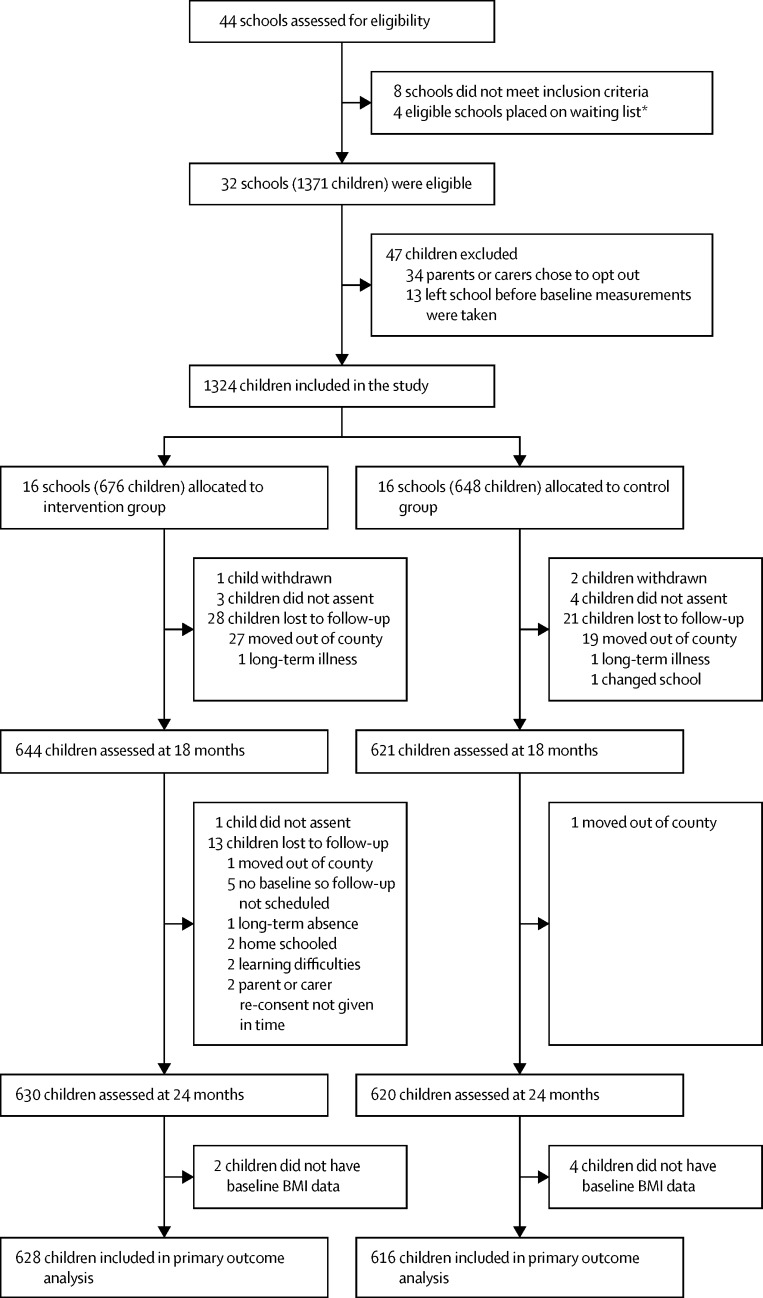
Trial profile *Two schools that had been allocated to cohort 2 withdrew while waiting to commence the trial and so were replaced with two of the four schools on the waiting list before cohort 2 commenced the trial. All schools that started the trial remained in the trial and so all the randomised clusters are present at baseline and at each follow-up point. BMI=body-mass index.

Between March 21, 2012, and Sept 30, 2013, 36 eligible schools were identified, of which four were placed on the waiting list. We recruited 32 schools with 1371 eligible children, of whom 1324 participated in the study (figure 1). 16 schools (676 children) were assigned to the intervention group and 16 schools (648 children) to the control group. We compared characteristics of the primary schools in the HeLP trial with other primary schools in Devon and England (appendix p 2). HeLP schools had a similar average number of pupils, deprivation, and academic achievement to English schools; however, the proportion of pupils with English as a second language was significantly lower than the national average (4·1% in HeLP schools *vs* 16·8% in all schools in England), although it was nearly double the proportion in Devon schools as a whole, which is 2·6%. The intervention and control groups had similar school-level and child-level baseline characteristics, including physical activity and food intake questionnaire scores (table 1). At baseline, although anthropometric measurements between the groups were largely similar, a greater proportion of children in the intervention group were overweight or obese than in the control group (table 1).

**Table 1:** Baseline characteristics of participating schools and children

		**Intervention group**	**Control group**
**Cluster level**			
Number of schools		16	16
Number of participating children per school		35·4 (26·5–50·0)	33·5 (28·5–51·0)
School IMD*		14 380 (12 806–21 446)	13 341 (9208–21 785)
Number of year-5 classes			
	Single class	8 (50%)	9 (56%)
	More than one class	8 (50%)	7 (44%)
Free school meals			
	<19% of pupils	9 (56%)	9 (56%)
	≥19% of pupils	7 (44%)	7 (44%)
Cohort			
	Cohort 1	8 (50%)	8 (50%)
	Cohort 2	8 (50%)	8 (50%)
**Individual level**			
Number of children		676	648
Age, years		9·8 (0·3)	9·7 (0·3)
Sex			
	Female	336 (50%)	343 (53%)
	Male	340 (50%)	305 (47%)
Child IMD*		16 060 (12347–21957)	13 171 (6741–20 882)
BMI SDS		0·32 (1·16)	0·18 (1·14)
Waist circumference SDS		0·72 (1·11)	0·55 (1·15)
Percentage body fat SDS		−0·61 (2·18)	−0·63 (2·38)
Percentage body fat SDS (excluding extreme body fat)†		−0·39 (1·62)	−0·46 (1·52)
Weight status‡			
	Underweight	11 (2%)	10 (2%)
	Healthy	479 (72%)	483 (75%)
	Overweight	81 (12%)	69 (11%)
	Obese	98 (15%)	81 (13%)
	Missing data	7 (1%)	5 (1%)
Physical activity§			
	Weekly acceleration, mg	49·0 (11·3)	49·6 (10·9)
	Daily total, min	182·7 (36·7)	185·0 (34·7)
	Daily light, min	129·4 (24·7)	131·1 (24·2)
	Daily moderate, min	40·0 (12·1)	40·4 (11·4)
	Daily moderate to vigorous, min	53·3 (16·8)	53·9 (16·2)
	Daily vigorous, min	13·3 (6·2)	13·5 (6·2)
	Daily sedentary, min	780·4 (36·1)	778·2 (34·0)
Food intake questionnaire scores, all days of the week			
	Daily energy-dense snacks	4·2 (2·2)	4·1 (2·2)
	Daily healthy snacks	3·3 (1·6)	3·1 (1·6)
	Daily positive food markers	6·0 (2·7)	5·7 (2·5)
	Daily negative food markers	6·8 (3·4)	6·8 (3·3)
Food intake questionnaire scores, weekdays			
	Daily energy-dense snacks	4·0 (2·4)	4·0 (2·4)
	Daily healthy snacks	3·4 (1·8)	3·2 (1·7)
	Daily positive food markers	6·1 (2·9)	5·7 (2·8)
	Daily negative food markers	6·5 (3·7)	6·7 (3·8)
Food intake questionnaire scores, weekend days			
	Daily energy-dense snacks	4·6 (2·5)	4·4 (2·4)
	Daily healthy snacks	3·2 (1·9)	2·9 (1·8)
	Daily positive food markers	6·0 (3·1)	5·5 (2·9)
	Daily negative food markers	7·7 (4·0)	7·1 (3·6)

Data are n (%), mean (SD), or median (IQR). IMD=index of multiple deprivation. BMI=body-mass index. SDS=standard deviation score.

* School IMD is related to the school's postcode and child IMD is related to child's home postcode.^18^

† After excluding extreme body fat absolute SDS ≥5.

‡ At baseline, height and weight measurements were available for 669 (99%) of 676 children in the intervention and 643 (99%) of 648 in the control group. Weight status categories defined using the Public Health England definitions^12^ (underweight ≤2nd UK National BMI percentile relevant to the UK 1990 reference data, healthy >2nd and <85th BMI percentile, overweight ≥85th and <95th BMI percentile, and obese ≥95th BMI percentile).

§ n=428 in intervention group and n=458 in control group.

All 32 schools completed the trial. All schools in the intervention group completed or nearly completed the whole programme and the quality of delivery in all schools was at or above the established appropriate level (appendix). 629 (93%) of the 676 children in the intervention group were categorised as compliers (ie, they received at least four of the five drama sessions and the one-to-one goal-setting discussion in phase 3). No notable differences in uptake were seen between the two cohorts (appendix). 353 (52%) of the 676 children had family attending at least one parent event and 652 (96%) children set goals with the HeLP coordinator in phase 3. 411 (63%) of these 652 children had parental support, shown by a parent signature on the goal-setting sheet or written comments about how the parent would support the child in achieving their goals.

1244 children were included in the primary analysis of BMI SDS because both baseline and 24-month BMI data were available for them (figure 1). In the measurement training sessions before anthropometric measures were taken, inter-rater reliability for height and waist circumference was high (coefficients of variations were, respectively, 0·2% and 1·3% at baseline, 0·1% and 1·2% at 18 months, and 0·1% and 0·4% at 24 months). No child had reported his or her allocated group to the masked assessor at 24-month follow-up. Of the 886 children who wore accelerometers, 851 (96%) had usable physical activity data files (ie, files could be downloaded and were not corrupted) at baseline and 788 (89%) had usable data at 18 months; similarly, the number of children with valid physical activity data after the application of the minimum wear requirements (three weekdays and one weekend day with a minimum of 10 h of wear time per day) was 830 (94%) at baseline and 745 (84%) at 18 months. 701 (79%) of the 886 children achieved the full 7 days of 10 h wear time per day. No evidence suggested differences between the groups in terms of the completeness of outcome measures throughout the trial, although more children in the intervention group were lost to follow-up than in the control group (figure 1). No differences were noted between control and intervention schools at either baseline or 18 months in terms of the number and type of school nutrition and physical activity policies they had in place (data not shown).

Mean BMI SDS at 24 months was 0·35 (SD 1·25) in children in the intervention group and 0·22 (1·22) in those in the control group (table 2). With adjustment for school-level clustering, baseline BMI scores, sex, cohort, and number of year-5 classes and socioeconomic status of each school, the mean difference in BMI SDS score (intervention–control) at 24 months was −0·02 (95% CI −0·09 to 0·05, p=0.57).

**Table 2:** Primary and secondary anthropometric outcomes at 18 and 24 months

		**Intervention group**		**Control group**		**Mean difference (intervention–control) or odds ratio (95% CI)**		p value*
		N†	Mean (SD) or (%)	N†	Mean (SD) or (%)	Adjusted for clustering only	Fully adjusted‡	
**18 months**								
BMI SDS		644	0·32 (1·23)	621	0·20 (1·23)	0·11 (−0·12 to 0·33)	−0·02 (−0·08 to 0·05)	0·61
Waist circumference SDS		645††	0·69 (1·18)	620§	0·57 (1·15)	0·08 (−0·15 to 0·32)	−0·07 (−0·27 to 0·12)	0·44
Percentage body fat SDS								
	All children	644	−0·99 (2·23)	619§	−0·98 (2·03)	−0·02 (−0·38 to 0·35)	−0·02 (−0·25 to 0·22)	0·90
	After exclusion of extreme values¶	618	−0·74 (1·84)	593	−0·75 (1·73)	0·01 (−0·29 to 0·31)	−0·02 (−0·16 to 0·12)	0·77
Weight status||								
	Underweight and healthy weight	458	71%	463	75%	NA	NA	NA
	Overweight	87	14%	78	13%	NA	NA	NA
	Obese	99	15%	80	13%	NA	NA	NA
	Overweight and obese	186	29%	158	25%	1·18** (0·80 to 1·72)	1·05** (0·58 to 1·88)	0·88
**24 months**								
BMI SDS		630	0·35 (1·25)	620	0·22 (1·22)	0·11 (−0·11 to 0·33)	−0·02 (−0·09 to 0·05)	0·57
Waist circumference SDS		629§	0·63 (1·24)	618§	0·54 (1·21)	0·09 (−0·15 to 0·33)	−0·05 (−0·23 to 0·13)	0·56
Percentage body fat SDS								
	All children	629§	−0·78 (2·16)	620	−0·78 (1·89)	−0·02 (−0·37 to 0·33)	−0·04 (−0·29 to 0·22)	0·76
	After exclusion of extreme values¶	612	−0·59 (1·84)	607	−0·65 (1·69)	0·04 (−0·23 to 0·32)	−0·02 (−0·17 to 0·13)	0·79
Weight status||								
	Underweight and healthy weight	436	69%	455	73%	NA	NA	NA
	Overweight	89	14%	84	14%	NA	NA	NA
	Obese	105	17%	81	13%	NA	NA	NA
	Overweight and obese	194	31%	165	27%	1·19** (0·82 to 1·71)	1·09** (0·70 to 1·69)	0·72

BMI=body-mass index. SDS=standard deviation score. NA=not applicable; the logistic regression to produce odd ratios was only applicable to the combined overweight and obese category, dichotomised into levels of overweight and obese versus normal and underweight.

* Fully adjusted mean difference.

† N is the total number of children from whom we collected data at that timepoint.

‡ Estimated using random-effects linear or logistic regression models (comparing overweight or obese with underweight or healthy weight) to account for clustering among children within the same school, with adjustment for stratification variables (number of year-5 classes and proportion of children eligible for free school meals), cohort, sex, and baseline measure of outcome under consideration.

§ Some data for some children were not collected because they were absent on days of assessment or they left or moved between schools.

¶ After excluding extreme body fat absolute SD values ≥5.

|| Weight status categories defined using the Public Health England definitions^12^ (underweight ≤2nd UK National BMI percentile relevant to the UK 1990 reference data, healthy >2nd and <85th BMI percentile, overweight ≥85th and <95th BMI percentile, and obese ≥95th BMI percentile).

** Results from logistic regression analysis.

†† At 18 months, one child had waist circumference measurement but no weight measurement, so BMI could not be calculated.

**Figure 2:**
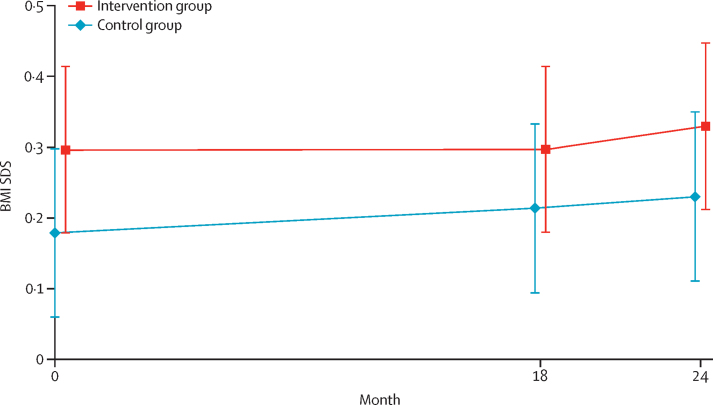
Predicted marginal BMI SDS with 95% CIs in the two groups across timepoints Data are derived from the repeated measures, allowing for hierarchical clustering by child within each school, modelling the within-child covariance between fixed timepoints as an autoregressive pattern of order one. BMI=body-mass index. SDS=standard deviation score.

According to the repeated measures model, no significant difference in mean BMI SDS existed between the two allocated groups at baseline (0·30 [95% CI 0·18 to 0·41] in the intervention group and 0·18 [0·06 to 0·30] in the control group, p=0·17; figure 2). The BMI SDS predicted by the model was 0·30 (95% CI 0·18 to 0·41) in the intervention group and 0·21 (0·09 to 0·33) in the control group at 18 months, increasing to 0·33 (0·21 to 0·45) in the intervention group and 0·23 (95% CI 0·11 to 0·35) in the control group by 24 months (figure 2). The sensitivity analyses to explore assumptions about missing primary outcome data produced results that were consistent with the primary analysis (appendix). We found no evidence that the intervention effect was modified in any of the prespecified subgroups (appendix).

No significant differences were seen between the groups in any of the other anthropometric outcomes at either 18 or 24 months (table 2), nor any of the physical activity outcomes at 18 months (table 3). The adjusted means of FIQ scores (both weekly and weekday) for energy-dense snacks and negative food markers were lower in the intervention group than in the control group (table 4). The discrete values of the weekday and weekend scores, bounded by zero, suggested that the assumptions for modelling in a linear model might not be fully met, despite the apparent symmetrical, normal shape of the data in the plots. Therefore, random-effects ordinal logistic regression models were also fitted to these outcomes. A positive effect of the intervention was still seen on the weekday scores for energy-dense snacks and negative food markers (data not shown); however, the p values are close to 0·05 and the difference could be due to chance.

**Table 3:** Primary intention-to-treat analyses of secondary physical activity outcome measures assessed at 18 months after baseline

	**Intervention group (n=359)**	**Control group (n=386)**	**Mean difference (intervention–control) (95% CI)**		**p value**†
			Adjusted for clustering only	Fully adjusted*	
Weekly acceleration, mg	52·14 (13·95)	51·47 (12·95)	0·53 (−2·18 to 3·24)	0·57 (−1·58 to 2·72)	0·59
Daily total, min	199·71 (43·94)	198·05 (40·20)	1·23 (−8·24 to 10·70)	1·26 (−6·84 to 9·36)	0·75
Daily light, min	141·72 (27·80)	141·07 (27·09)	0·43 (−5·87 to 6·73)	0·70 (−4·73 to 6·13)	0·79
Daily moderate, min	44·26 (16·24)	43·46 (13·43)	0·68 (−2·41 to 3·78)	0·41 (−2·28 to 3·09)	0·76
Daily moderate to vigorous, min	57·99 (22·34)	56·98 (19·39)	0·85 (−3·24 to 4·94)	0·56 (−2·76 to 3·89)	0·73
Daily vigorous, min	13·73 (7·66)	13·52 (7·38)	0·15 (−1·33 to 1·63)	0·15 (−1·01 to 1·3)	0·80
Daily sedentary, min	764·50 (43·29)	766·36 (39·88)	−1·46 (−10·91 to 8·00)	−1·39 (−9·45 to 6·68)	0·73

Data are mean (SD) unless specified otherwise.

* Estimated using random-effects linear regression models to account for clustering among children within the same school, with adjustment for stratification variables (number of year-5 classes and proportion of children eligible for free school meals), cohort, sex, and baseline measure of the outcome under consideration.

† Fully adjusted mean difference.

**Table 4:** Food intake questionnaire outcomes at 18 months

	**Intervention group**		**Control group**		**Mean difference (intervention–control) (95% CI)**		**p value**†
	N	Mean (SD)	N	Mean (SD)	Adjusted for clustering only	Fully adjusted*	

**Weekly food intake questionnaire scores**							
Daily energy-dense snacks	646	3·72 (1·86)	624	4·06 (2·07)	−0·29 (−0·64 to 0·06)	−0·37 (−0·66 to −0·07)	0·017
Daily healthy snacks	637	3·61 (1·63)	617	3·30 (1·50)	0·31 (0·02 to 0·60)	0·22 (−0·04 to 0·47)	0·092
Daily negative food markers	647	5·90 (2·73)	624	6·38 (3·00)	−0·40 (−0·94 to 0·14)	−0·47 (−0·91 to −0·02)	0·041
Daily positive food markers	647	6·20 (2·36)	624	5·77 (2·31)	0·42 (0·01 to 0·84)	0·26 (−0·12 to 0·64)	0·17
**Weekday food intake questionnaire scores**							
Daily energy-dense snacks	647	3·54 (2·03)	625	3·99 (2·27)	−0·41 (−0·83 to 0·01)	−0·47 (−0·84 to −0·11)	0·013
Daily healthy snacks	645	3·69 (1·77)	624	3·38 (1·64)	0·30 (−0·04 to 0·64)	0·23 (−0·08 to 0·54)	0·14
Daily negative food markers	647	5·54 (2·94)	625	6·21 (3·28)	−0·61 (−1·25 to 0·03)	−0·64 (−1·17 to −0·11)	0·020
Daily positive food markers	647	6·28 (2·55)	625	5·87 (2·52)	0·39 (−0·09 to 0·86)	0·27 (−0·18 to 0·73)	0·23
**Weekend food intake questionnaire scores**							
Daily energy-dense snacks	647	4·17 (2·21)	626	4·26 (2·35)	0·01 (−0·37 to 0·37)	−0·10 (−0·43 to 0·24)	0·56
Daily healthy snacks	639	3·42 (1·83)	620	3·12 (1·73)	0·31 (0·02 to 0·59)	0·23 (−0·04 to 0·50)	0·086
Daily negative food markers	648	6·79 (3·24)	626	6·82 (3·36)	0·12 (−0·44 to 0·68)	−0·07 (−0·56 to 0·42)	0·77
Daily positive food markers	648	6·00 (2·66)	626	5·52 (2·64)	0·50 (0·05 to 0·95)	0·36 (−0·13 to 0·84)	0·15

* Estimated using random-effects linear regression models to account for clustering among children within the same school, with adjustment for stratification variables (number of year-5 classes and proportion of children eligible for free school meals), cohort, sex, and baseline measure of the outcome under consideration.

† Fully adjusted mean difference.

The intraclass correlation coefficient for BMI SDS at 24 months was 0·014 (95% CI 0·003–0·069; appendix p 12).

Three children withdrew from the trial (two from the control group and one from the intervention group), and one adverse event was reported by a concerned parent about her child's eating and activity behaviours (overexercising and restricting food intake). After discussion with the chief investigator, the parent was happy for their child to remain in the study and continue to participate in the intervention.

## Discussion

The risk of childhood obesity is related to a complex interaction of factors at the individual, family, school, and societal levels. The HeLP intervention was developed using intervention mapping based on previous evidence of effective approaches to modifying children's risk factors for obesity and creating supportive school and home environments for healthy behaviours; it was extensively piloted to ensure acceptability and feasibility.^10^, ^19^, ^20^ In this large school-based cluster randomised controlled trial we showed high fidelity to intervention delivery and participation by children and families, and successfully collected data on 84–96% of children for all outcome measures. We found no evidence of an intervention effect on the primary outcome of BMI SDS at 24 months, nor on any of the objectively measured anthropometric or physical activity outcomes. Based on self-report data, there was some weak evidence of a small but significant difference in favour of the intervention group in the mean number of different types of unhealthy snacks (energy dense) and unhealthy foods (negative markers) consumed.

Evidence from systematic reviews^5^, ^21^ suggests that some school-based intervention programmes that target physical activity and diet and involve activities to engage families have a modest effect on weight outcomes; however, the reviews identify significant between-study heterogeneity and acknowledge that most of the included studies have a moderate-to-high risk of bias. The most recent, methodologically rigorous, UK trial of a school-based intervention aimed at increasing physical activity, reducing sedentary behaviour, and increasing fruit and vegetable consumption in the same age group as HeLP, the Active for Life Year 5 trial,^22^ reported no difference between children in the intervention and control groups in the three primary outcomes (accelerometer-assessed moderate to vigorous physical activity, accelerometer-assessed sedentary activity, self-reported fruit and vegetable servings) or in the secondary outcome of weight status at 12 months.

In the HeLP trial, we aimed to address the methodological shortcomings identified in other studies and assess an intervention that included the behaviour change techniques believed most likely to affect identified causal pathways for obesity.^23^ It also aimed to engage children, families, and schools. We specifically sought to minimise key sources of bias, including recruitment, performance, and detection biases, by recruiting schools and children and collecting baseline measures before randomisation (to reduce differential uptake), capturing evidence of changes in school policies around food or physical activity during the trial, and using assessors who were masked to group allocation to measure the anthropometric outcomes. Although the FIQ was completed before revealing group allocation at baseline, the children were aware of their group allocation at the 18-month and 24-month follow-ups (appendix p 3). We also recognise that the HeLP coordinators collected measurements in both control and intervention schools, so contamination in the control schools might have occurred. However, the interaction between the coordinators and the control schools and children was minimal compared with that in the intervention schools, so taking the measurements probably did not affect obesity-related behaviours of the children to any great extent.

Sample size calculations, which allowed for the anticipated level of clustering as estimated from the exploratory trial and English National Child Measurement Programme (NCMP) data,^19^, ^24^ suggested that the trial needed outcome measures from 762 children at 24-month follow-up to detect a clinically meaningful difference in BMI SDS. However, the larger number of children per school than that anticipated, as well as successful trial recruitment and retention, meant that primary outcome data were available for 1250 children. Only a low risk of attrition bias existed in the study because few eligible children (34 [2%] of 1371) were opted out by their parents or carers and we achieved exceptional levels of follow-up at both 18 and 24 months for all outcome measures.

Reviews of school-based obesity prevention and obesity management trials in children have shown low participation, differential dropout, and high loss to follow-up.^5^, ^6^, ^21^, ^25^ For example, completeness of anthropometric data in school-based obesity prevention programmes has ranged from 70% to 80% for follow-up of 24 months or more,^26^, ^27^, ^28^ and the percentage of children providing a representative pattern of their physical activity levels across the entire week (at least three weekdays and one weekend day of 10 h wear time) tends to be much lower (40–60%).^16^ In the HeLP trial, 84% of children met this minimum wear time criteria and 79% provided data on 7 days, showing one of the most complete follow-ups and best compliance with physical activity assessment of obesity prevention trials in children of this age group. We attribute this to the extensive stakeholder involvement in the intervention development, trial design and delivery, and building of trusting and supportive relationships with schools, children, and families.^20^, ^29^

We weighted school recruitment to achieve a similar proportion of pupils eligible to receive free school meals as the national average, which is higher than the average for Devon (12·7%). 14 (44%) of the 32 participating schools had more than 19% pupils eligible for free school meals. Participating schools were larger than the average primary school in Devon, but in other respects, schools were representative of Devon and the anthropometric data from the children in the trial were broadly similar to the Devon NCMP year-6 data (no county-level data are available for year-5 classes because these measurements are taken in reception and year 6 only).^30^ The representative sample gives us confidence that the results are likely to be applicable to a wider population. We used the proportion of children with English as a second language as a proxy of ethnicity, and, although the included schools reflect the proportion of children from minority ethnic groups typical for Devon (6%), this value is substantially lower than the average proportion in England (28%; appendix p 2).^31^

We found that a theoretically informed complex intervention, which was feasible and acceptable to schools, children, and their families and achieved a high level of engagement, failed to change diet and physical activity behaviours and had no effect on weight status. Schools are ideal locations for childhood obesity prevention programmes given their near-universal reach of children across the socioeconomic spectrum; however, the capacity of such programmes to affect family behaviour patterns is poor. Children aged 9 and 10 years spend most of their time in either the school or family environment and it was these two environments we sought to affect, giving children the necessary skills to identify and make healthy diet and activity choices and engage their parents in supporting these behaviours. We gave the children autonomy to select which behaviours they wished to change and encouraged their families to identify how they would support their child to achieve their goals. However, the programme did not affect BMI SDS or physical activity, suggesting that we were unsuccessful in our overarching aim to affect both the school and family environments. Although HeLP used several activities to directly engage parents as well as activities to engage other year groups within the school, the programme did not explicitly seek to affect school policies or physical aspects of the school environment. Furthermore, in seeking to minimise the burden of delivery for schools, the use of external delivery personnel for much of the programme might have minimised any effect on school culture. However, we think schools are unlikely to find a more intensive programme feasible or acceptable to implement.

We believe that these findings, and results from other large, rigorous studies, call into question the likelihood that individually focused, school-based obesity prevention programmes can ever be sufficient to reduce the risks of obesity in primary school children. In 2015, *The Lancet's* second Obesity Series called for an “urgent rethinking of the causes, enablers, and barriers to change” by focusing on the “reciprocal nature of the interaction between the environment and the individual”,^32^ in which feedback loops perpetuate food choices and behaviours. Schools have an important role in creating supportive social and physical environments; however, unless upstream determinants of obesity are also addressed, families are unlikely to feel supported or motivated to change their behaviours. Such whole-systems approaches to childhood obesity prevention are theoretically attractive, but both their practical application and evidence for their effectiveness are currently absent and will require rigorous investigation.
