# Supplementary material for: Effectiveness of the Healthy Lifestyles Programme (HeLP) to prevent obesity in UK primary-school children: a cluster randomised controlled trial
Source: Lancet Child Adolesc Health. 2018 Jan;2(1):35–45. doi: 10.1016/S2352-4642(17)30151-7 (PMC5738974; doi:10.1016/S2352-4642(17)30151-7)
Supplement: Supplementary appendix [file mmc1.pdf]

THE LANCET
Child & Adolescent Health

## Supplementary appendix

This appendix formed part of the original submission and has been peer reviewed. We post it as supplied by the authors.

Supplement to: Lloyd J, Creanor S, Logan S, et al. Effectiveness of the Healthy Lifestyles Programme (HeLP) to prevent obesity in UK primary-school children: a cluster randomised controlled trial. *Lancet Child Adolesc Health* 2017; published online Nov 28. http://dx.doi.org/10.1016/S2352-4642(17)30151-7.

HeLP

Healthy Lifestyles Programme

**The effectiveness of a novel Healthy Lifestyles Programme (HeLP) to prevent obesity in primary school children: a school-based cluster randomised controlled trial.**

**Web Appendix**


1. **Comparison of HeLP schools with primary schools in Devon and England**
2. **HeLP timeline cluster diagram**
3. **Intervention details**
4. **Details of the timing and methods of capture of the outcome measures at 18 and 24 months post baseline**
5. **Uptake of HeLP across the four phases for each cohort**
6. **Fidelity of Intervention**
7. **Sensitivity analyses of BMI SDS (primary outcome) assessed at 24 months post baseline**
8. **Primary intention to treat analyses of BMI SDS (primary outcome) assessed at 24 months post baseline in pre-specified sub-groups**
9. **Estimates of the intra-class correlation coefficients from the random effects models for outcomes at 18 and 24 months post baseline**
10. **Food Intake Questionnaire scoring**
11. **References**

1. **Table A1: Comparison of HeLP schools with primary schools in Devon and England**

| **Characteristics** | **HeLP Schools** | **Devon Schools** | **Schools in England [1]** |
|---|---|---|---|
| % of children eligible for free school meals | 20 | 12·7 | 19 |
| Average number pupils/ school | 306 | 168 | 251 |
| % of children achieving Level 4 at Key Stage 2 | 79 | 81 | 81 |
| Proportion of pupils with English as an Additional Language | 4·1 | 2·6 | 16·8 |

**2. Figure A1: HeLP timeline cluster diagram**

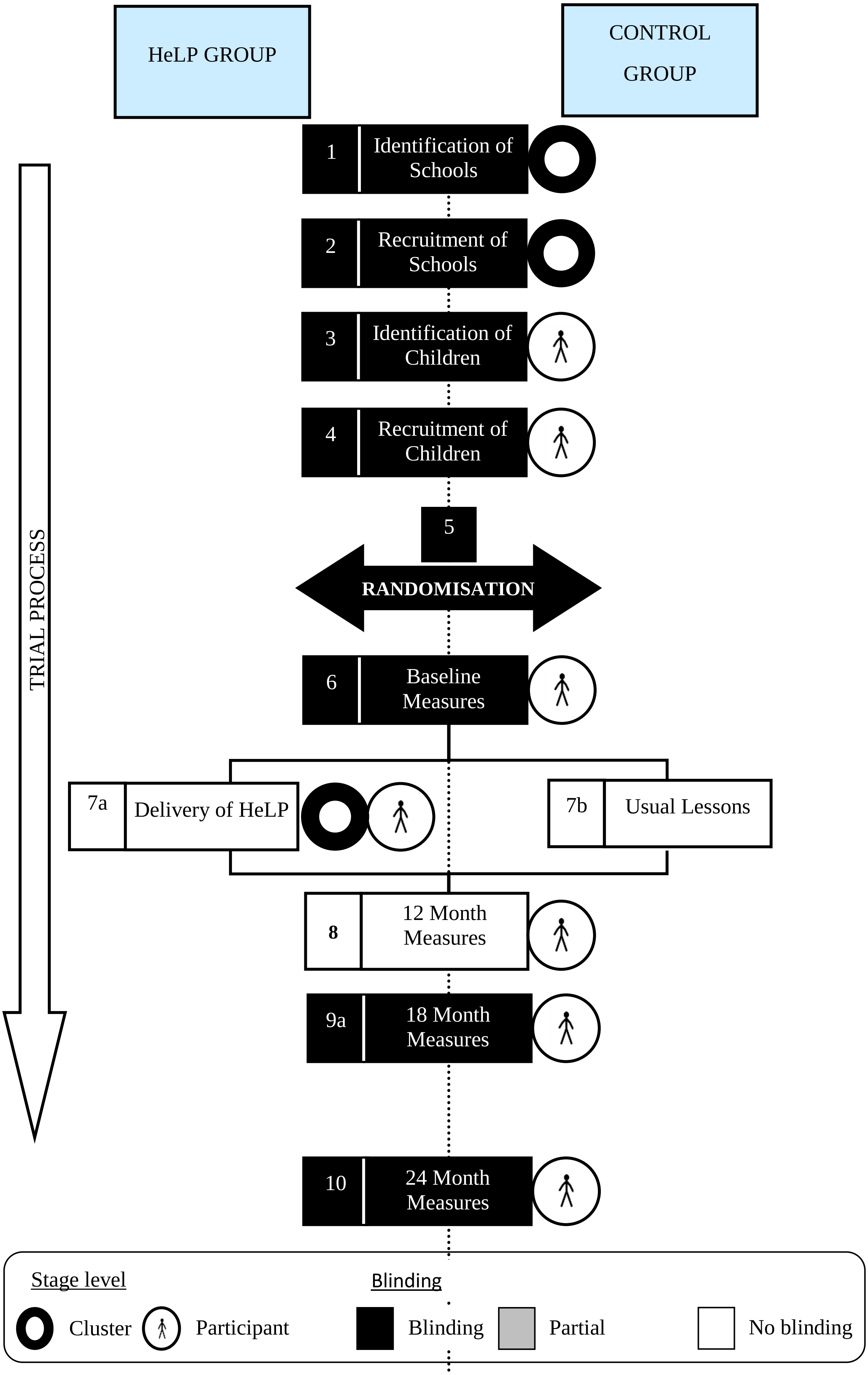

Key for the HeLP timeline cluster diagram[2]

| | |
|---|---|
| 1 | *Identification of schools*<br>Trial team attended Devon primary heads' meetings and network events |
| 2 | *Recruitment of schools*<br>All Devon state primary and junior schools with children in at least one single Year 5 group of ≥20 children eligible to participate. Schools that expressed interest in participating were purposely sampled to represent a range of number of Year 5 classes (1-3 Year 5 classes), locations (urban and rural), and deprivation (<19% and ≥19% of children eligible for free school meals). Remaining schools placed on waiting list for Cohort 2 (two schools allocated to Cohort 2 dropped out before commencing trial and replaced by a school from the waiting list) |
| 3 | *Identification of children*<br>All children in Year 5 within each recruited school eligible to participate |
| 4 | *Recruitment of children*<br>Information sent to Year 5 child's parent/carer. Opportunity to opt out child from trial |
| 5 | *Randomisation*<br>Allocation of schools to intervention or control group was using a computer-generated sequence, stratified on School Size (one Year 5 class vs ≥2 Year 5 classes) and % of pupils eligible for Free School Meals (<19%, ≥19%), as an indicator of school-level socioeconomic status. After randomisation to intervention or control, schools were allocated to Cohort 1 or Cohort 2 by a statistician from the CTU, with equal numbers of intervention and control schools in both cohorts. Allocation remained concealed from all delivery personnel, schools, and children until after baseline measures were captured in each cohort |
| 6 | *Baseline measures*<br>Performed by trained assessors, who were blinded to schools' allocated groups |
| 7a | *Intervention delivery*<br>HeLP intervention delivered to schools and children allocated to the intervention group. No blinding for schools, HeLP coordinators, children, or parents/carers |
| 7b | *Usual care*<br>Children in schools allocated to control group receive usual care. No blinding for schools, HeLP coordinators, children, or parents/carers |
| 8 | *12 month measure*<br>Self-completed My Lifestyles Questionnaire |
| 9a | *18 month measures*<br>Anthropometric measures collected by trained independent assessors blinded to allocated group |
| 9b | *18 month measures*<br>Self-completed Food Intake Questionnaire, whilst children still at primary school, 18 months post baseline. Physical activity data from a subset of children, whilst children still at primary school, 18 months post baseline, was objectively assessed |
| 10 | *24 month measures*<br>Anthropometric measures collected by trained independent assessors blinded to allocated group, after children have moved to secondary school (secondary schools have mix of children from intervention and control schools), 24 months post baseline (12 months post intervention) |

**3. Intervention details**

We used an Intervention Mapping (IM)[3] approach in developing the intervention, which involved firstly conducting a literature review and extensive stakeholder consultation (step 1 of the intervention mapping process), followed by developing a logic model as recommended,[3] specifying programme objectives and their associated behavioural and environmental determinants (step 2), selecting theory-based techniques capable of changing behaviour patterns[4,5] and selecting context-appropriate delivery methods (step 3) then designing and producing intervention components and delivery materials (step 4). Steps 5 and 6 of the IM process involved assessing the feasibility and acceptability of the newly-developed intervention in a number of pilot studies.[6-8]

The IM approach identified two theoretical frameworks within which changes in behaviour patterns and their determinants were conceptualised: (a) the Information, Motivation and Behavioural Skills Model (IMB)[9] which linked to the selected determinants of the key behavioural objectives for the programme (to replace unhealthy snacks and drinks with healthy alternatives and to replace screen time with physical activity) and (b) the Health Action Process Model (HAPA),[10] which guided the sequential order in which to deliver the theory-based behaviour change techniques. The HAPA model implies a clear order of distinct actions starting with establishing motivation, moving on to taking action, followed by maintaining motivation.

The intervention consists of four phases delivered over three school terms, which have been ordered to enable and support behaviour change (see Table A2). The programme delivers a general healthy lifestyle message, encouraging a healthy energy balance with a focus on behaviours relating the consumption of sweetened fizzy drinks; healthy and unhealthy snacks, physical activity, and reducing screen time. An overarching message promoted throughout the Healthy Lifestyles Programme (HeLP) is the ‘80/20’ rule, which recommends we should eat healthily and be active at least 80% of the time. Phases 1-3 of the intervention takes place when the children are in school Year 5 (aged 9-10 years) and phase 4 takes place at the beginning of school Year 6 (aged 10-11 years). The intervention was designed to fit in with the National Curriculum at Key Stage 2 and all lessons and drama sessions include learning objectives relating to Personal Social and Health Education (PSHE), Science, Numeracy, and Literacy.

**Table A2: Behaviour change techniques (BCTs), components and delivery personnel by intervention phase**

| Phase | Behaviour Change techniques (BCTs) | Component (Frequency and Duration) | Delivery personnel |
|---|---|---|---|
| **Phase 1**<br><br>**Creating a supportive context**<br><br>Spring term<br><br>(Yr 5) Jan-March | • Provide information on behaviour-health link<br>• Provide information on health behaviour link<br>• Modelling/demonstrating behaviour<br>• Prompt identification as a role model<br>• Provide information on behaviour-health link<br>• Skill building | Whole school assembly (1x20 mins)<br><br>Newsletter article<br><br>Literacy lesson (to create HeLP rap) (1x1 hour)<br><br>Activity workshops (2x1·5 hours)[b]<br><br>Parent assembly (1x1 hour) involving child performances[a] | HeLP Coordinators<br><br>HeLP Coordinators<br><br>Class teacher<br><br>Professional sportsmen/dancers<br><br>Class teachers/HeLP Coordinator/Drama group |
| **Phase 2**<br><br>**Intensive Healthy Lifestyles Week –** one week<br><br>Summer term (Yr 5) April-June | • Provide information on health behaviour link<br>• Problem solving/barrier identification<br>• Modelling/demonstrating behaviour<br>• Prompt identification as a role model<br>• Communication skills training<br>• Teach to use prompts/cues | Education lessons (5x1 hour) (morning)<br><br>Drama (5x2 hours) (afternoon)[b]<br><br>(forum theatre; role play; food tasting, discussions, games, etc). | Class teacher<br><br>Drama group |
| **Phase 3**<br><br>**Personal Goal Setting with Parental Support**<br><br>Summer term (Yr 5) June-July | • Self-monitoring<br>• Goal setting (behaviour)<br>• Problem solving/barrier identification<br>• Plan social support<br>• Provide information on where and when to perform a behaviour<br>• Agree behavioural contract<br>• Prompt identification as a role model | Self-reflection questionnaire (1x40 mins)<br><br>Goal setting sheet to go home to parents to complete with child (1x10 mins)[c]<br><br>1:1 goal setting interview (1x10 mins) (goals sent home to parents)<br><br>Forum theatre assembly (1x1 hour)[a] | HeLP Coordinator<br><br>HeLP Coordinator/Parents<br><br>HeLP Coordinator<br><br>HeLP Coordinator/Drama group |
| **Phase 4**<br><br>**Reinforcement Activities**<br><br>Autumn term<br><br>(Yr 6) Sept-Dec | • Provide information on health behaviour link<br>• Modelling/demonstrating behaviour<br>• Prompt identification as a role model<br>• Provide social approval<br>• Prompt self-monitoring<br>• Prompt intention formation<br>• Follow up prompts<br>• Prompt review of behavioural goals<br>• Prompt barrier identification and resolution<br>• Coping plans | Education lesson (1x1 hour)<br><br>Drama workshop (1x1 hour). Followed by a class delivered assembly about the project to rest of school (1x20 mins)<br><br>1-to-1 goal supporting interview to discuss facilitators/barriers and to plan new coping strategies (1x10 mins) (renewed goals sent home to parents) | Class teacher<br><br>Drama group<br><br>HeLP Coordinator<br><br>HeLP Coordinator |

[a] Formal parental engagement event
[b] Invitation for parents to observe
[c] Required parental involvement to complete task

*Delivery personnel*

HeLP Coordinators coordinated the collection of measurements and the delivery of HeLP in schools (intervention and control schools). HeLP Coordinators were responsible for delivering components of the Programme and building relationships with schools, children and families. A Drama Facilitator led the drama sessions and coordinated delivery of the activities for each drama session in the Healthy Lifestyles Week (phase 2).

*Training for delivery personnel*

No training was required for the teachers to deliver the lessons and each lesson plan was set out clearly with objectives linking to the National Curriculum at Key Stage 2. Teachers were provided with a teaching manual containing all five lesson plans and associated resources in both hard and electronic form. All actors and the Drama Facilitator completed a four-day training programme and were given a detailed manual of the drama scripts and an overview (verbal presentation and written document) of the HeLP intervention. The Drama Facilitator for each drama team (comprising four actors) coordinated practise sessions prior to delivery in each school.

*Parental involvement*

Each phase of HeLP was designed to involve parents as much as possible. In phase 1, a newsletter was sent to parents and there was a parent assembly. In phase 2, an information leaflet was also sent home to parents each day based on the theme covered in the drama session and parents were invited in to the school to watch work in progress during the last two drama sessions of the week. In phase 3, children set goals at home with their parent/carer on a ‘goal setting’ sheet and returned to school with this sheet to discuss with the HeLP Coordinator. Finalised goals were then typed up and sent directly home to parents along with the HeLP ‘80/20’ fridge magnet as a further reminder of the Programme. Another parent assembly took place after the completed goals sheet had been sent home. In phase 4, following the one-to-one goal supporting session, a further sheet with the child’s goals was, once again, sent home in the post.

*Adaptability to local context*

HeLP was designed to allow for some flexibility, so that each activity could fit the context of the school. For example, schools were able to select the timings of parent assemblies and, throughout the year long intervention delivery period, the HeLP Coordinator worked closely with the teachers to understand how best to engage and involve the parents, which varied from school to school.

## 4. Table A3: Details of the timing and methods of capture of the outcome measures at 18 and 24 months post baseline*

| Outcome measure | Follow up time-point (s) | Month and Year | Equipment | Assessor | Procedure |
|---|---|---|---|---|---|
| Height | 18 and 24 months | June/July 2014/15 October/November 2014/15 | SECA stadiometer (Hamburg, Germany) | Independent assessor (blind to group allocation) | Height was recorded to an accuracy of 1mm. |
| Weight | 18 and 24 months | June/July 2014/15 October/November 2014/15 | Tanita Body Composition Analyser SC-330 (U.K. Ltd., Middlesex, U.K.) | Independent assessor (blind to group allocation) | Weight was recorded to within 0·1kg and children were asked to take off their shoes, socks and tights. |
| Waist circumference (WC) | 18 and 24 months | June/July 2014/15 October/November 2014/15 | Non-elastic flexible tape measure, 4cm above the umbilicus | Independent assessor (blind to group allocation) | WC was measured 4cm above the umbilicus. |
| Body Fat (BF) | 18 and 24 months | June/July 2014/15 October/November 2014/15 | Estimated from leg-to-leg bioelectric impedance analysis using the Tanita Body Composition Analyser SC-330 | Independent assessor (blind to group allocation) | All anthropometric measures (height, weight, waist circumference, and percentage body fat) were collected over the course of one day in each school.<br><br>If children were absent on the day of measurement, up to three further attempts to collect their data were made up to a further two weeks from the day of absence. All assessors completed refresher training prior to each data collection time-point. |
| Food intake (weekday and weekend) | 18 months | June/July 2014/15 | Adapted version of the validated Food Intake Questionnaire for weekdays and for the weekend (FIQ)[11]<br><br>Provides an estimation of the number of different types of healthy and unhealthy food and drink items consumed per day.<br><br>The questionnaire consisted of 10 healthy and 13 unhealthy snacks and drinks and 25 negative and 22 positive food markers. | HeLP Coordinator | Children answer yes or no as to whether they had consumed each listed food item the previous day.<br><br>The FIQ weekday was completed on either Tuesday Wednesday, Thursday, or Friday and the FIQ weekend was completed on a Monday.<br><br>Children were arranged in literacy groups to ensure that assistance could be given as efficiently as possible. An additional researcher, the class teacher and the teaching assistant also provided support. |
| Physical Activity | 18 months | June/July 2014/15 | Wrist worn waterproof tri-axial accelerometer called the GeneActiv[12]<br><br>PA can measure between +/- 8m*g* at a rate of up to 100Hz<br><br>One class per participating school was randomly selected for the physical activity data collection. If the school only had one class then that class was selected to wear the monitor. | HeLP Coordinator | Children were asked to wear the accelerometer continuously (including at night) for eight consecutive days on the wrist of the non-dominant arm. Information packs were sent to parents one week prior to children being fitted with the GeneActivs providing information on wearing the accelerometer and guidance to be distributed to sports coaches to prevent removal during sport. On the day the accelerometers were issued, the HeLP Coordinator spoke to ten children at a time about how to comply with the procedures and answered any questions. |

*all measures also collected at baseline (prior to randomisation)

## 5. Table A4: Uptake of HeLP across the four phases for each cohort

| | Phase 1 | Phase 2 | Phase 3 | Phase 4 | Percentage of children receiving 4 drama sessions and the goal setting* delivered in the spirit of HeLP** |
|---|---|---|---|---|---|
| **Number of components** | 5 | 10 | 2 | 4 | 5 |
| **Cohort 1 (n = 254)** | 91·2% (1158/1270) | 94·1% (2391/2540) | 91·1% (463/508) | 92·1% (936/1016) | 93·7% (238/254) |
| **Cohort 2 (n = 422)** | 94·7% (1998/2110) | 93·7% (3954/4220) | 92·5% (781/844) | 91·4% (1542/1688) | 92·7% (391/422) |
| **Total** | 93·4% (3156/3380) | 93·9% (6345/6760) | 92·0% (1244/1352) | 91·6% (2478/2704) | 93·0% (629/676) |

*Dose of HeLP deemed to be essential for behaviour change
***Enthusiastic delivery, open body language, responsive to child/school needs and clear and friendly communication*

## 6. Fidelity of Intervention

*Adherence to intervention components*

Adherence was assessed using yes/no checklists to indicate whether subcomponents within each component had been delivered or not. These were completed by the HeLP Coordinator or the Year 5 class teacher (for teacher delivered components only). Teacher completed checklists were returned to the HeLP Coordinator. If all components for each phase were delivered as per the manual (represented as a tick on the checklist) then it was recorded that 100% of HeLP components had been delivered in that school. If a minor activity (e.g. the practise of a chant, a missed scene from a drama workshop) was missing from a particular component, 2% was deducted for each minor activity from the overall total.

*Quality of intervention delivery*

Four components were chosen to assess quality of intervention delivery (one in each phase). These were the parent assembly (phase 1), the Healthy Lifestyles Week (phase 2), the parent assembly (phase 3) and the class delivered assembly (phase 4). For each of the four components, a score between 1 and 10 was given for (i) delivery; (ii) child response; (iii) parent response and (iv) teacher response. The criteria used to assess the quality of delivery were enthusiasm, open body language, responsivity to child/school needs and clear and friendly communication. The criteria to assess participant response to the delivery were attentiveness, positive body language (e.g. smiling, open posture) and active involvement (when required). These criteria evolved from the extensive piloting of the programme and in discussion with the advisory group and were felt to encompass the 'spirit' of the Healthy Lifestyles Programme.

The Trial Manager and the Principal Investigator independently scored quality of intervention delivery for the parent assembly in phase 1, across three schools. No discrepancy in scoring was observed for either quality of delivery or participant response. Thereafter, the majority of the observations were carried out by the Trial

Manager. The HeLP Coordinators assessed all the five drama sessions in the Healthy Lifestyle Week components (phase 2), after having carried out initial assessments alongside the Trial Manager.

The four scores (quality of delivery and child, parent and teacher response) per observation in each school were averaged (mean score) to create a single summary score out of 10 for each of the four components observed. These four component scores were then averaged to produce a single mean delivery score per school. A score ≥ 8 was pre-specified to indicate that the intervention had been delivered in the 'spirit' in which it had been designed.

*Participant responsiveness to the intervention overall*

Levels of engagement with the programme overall were assessed both quantitatively and qualitatively for schools and for individual Year 5 teachers and children in intervention schools.

**7. Table A5: Sensitivity analyses of BMI SDS (primary outcome) assessed at 24 months post baseline**

| | **Intervention Group** | | **Control Group** | | **Total n in analysis** | **Fully adjusted[a] mean difference in means (intervention – control) (95% CI)** | **P-value** |
|---|---|---|---|---|---|---|---|
| | **n** | **Mean (SD)** | **n** | **Mean (SD)** | | | |
| ***Random effects model*** | | | | | | | |
| Complete case data | 630 | 0·35 (1·25) | 620 | 0·22 (1·22) | 1244 | -0·02 (-0·09 to 0·05) | 0·57 |
| Worst-case imputation[b] | 671 | 0·43 (1·25) | 647 | 0·23 (1·23) | 1312 | 0·07 (-0·02 to 0·16) | 0·13 |
| Best-case imputation[b] | 671 | 0·34 (1·24) | 647 | 0·23 (1·23) | 1312 | -0·02 (-0·09 to 0·05) | 0·49 |
| ***Generalised estimating equation model*** | | | | | | | |
| Complete case data | 630 | 0·35 (1·25) | 620 | 0·22 (1·22) | 1244 | -0·02 (-0·08 to 0·05) | 0·57 |

[a] estimated using random effects linear regression models or generalised estimating equation model to account for clustering among children within the same school, with adjustment for stratification variables (number of Year 5 classes and proportion of children eligible for free school meals), cohort, gender, and baseline measure of outcome under consideration

[b] Missing BMI SDS at 24 months were imputed for all children with a baseline BMI SDS (n=1312)

**8. Table A6: Primary intention to treat analyses of BMI SDS (primary outcome) assessed at 24 months post baseline in pre-specified sub-groups**

| | **HeLP Intervention Group** | | **Control Group** | | **Total n in analysis** | **Mean difference (intervention – control) or odds ratio (95% CI)** [a] | **P-value for interaction** [b] |
|---|---|---|---|---|---|---|---|
| **Sub-group** | **n** | **Mean (SD) or No. (%)** | **n** | **Mean (SD) or No. (%)** | | | |
| School size | | | | | | | |
| Single Year 5 class | 197 | 0·22 (1·28) | 249 | 0·11 (1·23) | 444 | -0·06 (-0·20 to 0·09) | 0·56 |
| Multiple Year 5 classes | 433 | 0·41 (1·23) | 371 | 0·29 (1·20) | 800 | -0·01 (-0·13 to 0·11) | |
| Free school meal category | | | | | | | |
| < 19% of children | 417 | 0·28 (1·21) | 359 | 0·05 (1·18) | 774 | -0·03 (-0·16 to 0·09) | 0·61 |
| ≥ 19% of children | 213 | 0·47 (1·30) | 261 | 0·44 (1·23) | 470 | 0·04 (-0·15 to 0·22) | |
| Gender | | | | | | | |
| Females | 316 | 0·33 (1·28) | 328 | 0·27 (1·20) | 642 | -0·01 (-0·09 to 0·08) | 0·85 |
| Males | 314 | 0·37 (1·21) | 292 | 0·15 (1·23) | 602 | -0·03 (-0·13 to 0·07) | |
| Cohort | | | | | | | |
| Cohort 1 | 237 | 0·33 (1·32) | 381 | 0·22 (1·21) | 615 | -0·01 (-0·18 to 0·17) | 0·57 |
| Cohort 2 | 393 | 0·36 (1·20) | 239 | 0·21 (1·24) | 629 | -0·06 (-0·20 to 0·08) | |
| Individual IMD | 630 | 0·35 (1·25) | 620 | 0·22 (1·22) | 1182 | 0·00 (-0·17 to 0·14) | 0·95 |
| Baseline BMI SDS | 630 | 0·35 (1·25) | 620 | 0·22 (1·22) | 1244 | 0·00 (-0·09 to 0·05) | 0·95 |

[a] estimated using random effects linear regression models to account for clustering among children within the same school, with adjustment for stratification variables (number of Year 5 classes and proportion of children eligible for free school meals), cohort, gender, and baseline measure of outcome under consideration.
**[b]** p-values for the global test modelled as an interaction with the allocated group in the random effects model.

**9. Table A7: Estimates of the intra-class correlation coefficients from the random effects models for outcomes at 18 and 24 months post baseline**

| Outcome | ICC (95% CI) |
|---|---|
| **Primary outcome at 24 months** | |
| BMI SDS | 0·014 (0·003 to 0·069) |
| **Secondary outcomes at 24 months** | |
| Weight status[a] (overweight/obese vs. healthy/underweight) | 0·000 (0·000 to 1·000) |
| Waist circumference SDS | 0·116 (0·064 to 0·201) |
| Body fat SDS | 0·023 (0·007 to 0·075) |
| Body fat SDS[b] | 0·017 (0·003 to 0·078) |
| **Secondary outcomes at 18 months** | |
| *Anthropometric* | |
| BMI | 0·008 (0·001 to 0·081) |
| BMI SDS | 0·013 (0·003 to 0·064) |
| Weight status[a] (overweight/obese vs· healthy/underweight) | 0·060 (0·011 to 0·262) |
| Waist circumference SDS | 0·178 (0·105 to 0·285) |
| Body fat SDS | 0·012 (0·002 to 0·076) |
| Body fat SDS[b] | 0·012 (0·002 to 0·080) |
| *Physical activity* | |
| Mean weekly Acceleration (mg) | 0·053 (0·020 to 0·130) |
| Mean daily Total activity | 0·084 (0·039 to 0·174) |
| Mean daily Light | 0·095 (0·045 to 0·189) |
| Mean daily Moderate | 0·076 (0·034 to 0·163) |
| Mean daily Moderate-vigorous | 0·052 (0·020 to 0·130) |
| Mean daily Vigorous | 0·047 (0·017 to 0·123) |
| Mean daily Sedentary 6am-10pm | 0·085 (0·039 to 0·175) |
| *Food intake* | |
| Mean daily energy dense snacks | 0·023 (0·008 to 0·069) |
| Mean daily healthy snacks | 0·032 (0·012 to 0·083) |
| Mean daily negative marker foods | 0·026 (0·009 to 0·072) |
| Mean daily positive marker foods | 0·031 (0·012 to 0·080) |

[a] weight status categories are defined using the Public Health England definitions[13]
[b] after excluding extreme body fat absolute SD values greater than or equal to five

**10. Food Intake Questionnaire scoring**

**Table A8: Food Intake Questionnaire scoring for energy dense snack foods and healthy snack foods**

| **Energy dense snack foods (n=13)** | **FIQ Week item** | **FIQ weekend item** | **Healthy snack foods (n=10)** | **FIQ Week item** | **FIQ Weekend item** |
|---|---|---|---|---|---|
| High sugar cereals | **6** | **2** | Brown/wholemeal bread | **9** | **5** |
| Plain biscuits | **13** | **9** | Malt/fruit bread | **10** | **6** |
| Chocolate biscuits | **14** | **10** | Breadsticks/crackers | **11** | **7** |
| Cakes | **15** | **11** | Unsalted nuts | **34** | **30** |
| Puddings | **16** | **12** | Fresh fruit | **35** | **31** |
| Boiled sweets | **17** | **13** | Dried fruit | **36** | **32** |
| Chocolate | **18** | **14** | Salad | **37** | **33** |
| Ice cream | **19** | **15** | Hard cheese | **50** | **46** |
| Crisps | **27** | **23** | Yoghurt | **52** | **48** |
| Salty nuts | **33** | **29** | Semi skimmed milk | **59** | **55** |
| Pies/pasties | **44** | **40** | | | |
| Processed cheese | **51** | **47** | | | |
| Sweetened fizzy drinks | **55** | **51** | | | |

## 10. Food Intake Questionnaire scoring

**Table A9: Food Intake Questionnaire scoring for negative marker foods and positive marker foods**

| **Negative marker foods (n=25)** | **FIQ Week item** | **FIQ Weekend item** | **Positive marker foods (n=22)** | **FIQ Week item** | **FIQ Weekend item** |
|---|---|---|---|---|---|
| Sugar cereals | **6** | **2** | Low sugar cereals | **7** | **3** |
| Butter/margarine | **12** | **8** | Brown/wholemeal bread | **9** | **5** |
| Biscuits | **13** | **9** | Malt/fruit loaf | **10** | **6** |
| Chocolate biscuits | **14** | **10** | Breadsticks/crackers | **11** | **7** |
| Cakes | **15** | **11** | Boiled potatoes | **22** | **18** |
| Puddings | **16** | **12** | Mashed potatoes | **23** | **19** |
| Boiled sweets | **17** | **13** | Baked potatoes | **24** | **20** |
| Chocolate | **18** | **14** | Pasta | **28** | **24** |
| Ice cream | **19** | **15** | Rice | **29** | **25** |
| Sugar added to drinks | **20** | **16** | Noodles | **30** | **26** |
| Sugar added to food | **21** | **17** | Homemade pizza | **31** | **27** |
| Roast potatoes | **25** | **21** | Unsalted nuts | **34** | **30** |
| Crisps | **27** | **23** | Fresh fruit | **35** | **31** |
| Chips | **26** | **22** | Dried fruit | **36** | **32** |
| Salted nuts | **33** | **29** | Salad | **37** | **33** |
| Fried vegetables | **38** | **34** | Vegetables | **39** | **35** |
| Shop bought burger | **40** | **36** | Homemade burgers | **42** | **38** |
| Shop bought sausage | **41** | **37** | Homemade sausages | **43** | **39** |
| Pies and pasties | **44** | **40** | Yogurt | **52** | **48** |
| Fried fish | **46** | **42** | No sugar squash | **56** | **52** |
| Fried egg | **49** | **45** | Semi skimmed milk | **59** | **55** |
| Processed cheese | **51** | **47** | Water | **60** | **56** |
| Takeaways | **53** | **49** | | | |
| Salt added to food | **54** | **50** | | | |
| Sweet fizzy drink | **55** | **51** | | | |

## 11. References


1. UK Government. *Compare school and college performance*. Government Website. URL: https://www.compare-school-performance.service.gov.uk/download-data (Accessed 24 Oct 2016).
2. Caille A, Kerry S, Tavernier E, Leyrat C, Eldridge S, Giraudeau B. Timeline cluster: a graphical tool to identify risk of bias in cluster randomised trials. BMJ. 2016;354:i4291.
3. Bartholomew LK, Markham, C. M., Ruiter, R., Fernández, M. E,, Kok, G. & Parcel, G.S. . *Planning Health Promotion Programmes An Intervention mapping Approach (4th Ed)*; 2016.
4. Abraham C, Michie S. A taxonomy of behavior change techniques used in interventions. Health Psycholology. 2008;27(3):379-87.
5. Michie S, Ashford S, Sniehotta FF, Dombrowski SU, Bishop A, French DP. A refined taxonomy of behaviour change techniques to help people change their physical activity and healthy eating behaviours: the CALO-RE taxonomy. Psychol Health. 2011;26(11):1479-98.
6. Lloyd JJ, Logan S, Greaves CJ, Wyatt KM. Evidence, Theory and Context - Using intervention mapping to develop a school-based intervention to prevent obesity in children. Int J Behav Nutr Phys Act. 2011;8(1):73.
7. Wyatt KM, Lloyd JJ, Creanor S, Logan S. The development, feasibility and acceptability of a school-based obesity prevention programme: results from three phases of piloting. BMJ Open [Internet]. 2011 13 September 2011; 1(1):[e000026 p.]. Available from: http://bmjopen.bmj.com/content/1/1/e000026.
8. Lloyd JJ, Wyatt KM, Creanor S. Behavioural and weight status outcomes from an exploratory trial of the Healthy Lifestyles Programme (HeLP): a novel school-based obesity prevention programme. BMJ Open. 2012;2(3).
9. Fisher J, Fisher W. The information-motivation-behavioral skills model. In: DiClemente R, Crosby R, Kegler M, editors. *Emerging theories in health promotion practice and research*. San Fransisco: Jossy-Bass; 2002:40-70.
10. Schwarzer R. Self-efficacy in the adoption and maintenance of health behaviors: Theoretical approaches and a new model. In: R S, editor. *Self-efficacy: thought control of action*. London: Hemisphere; 1992:217-43.
11. Johnson B, Hackett A, Roundfield M, Coufopoulos A. An investigation of the validity and reliability of a food intake questionnaire. J Hum Nutr Diet. 2001;14(6):457-65.
12. ActivInsights Ltd. Kimboton U. URL: http://www.geneactiv.org/ (Accessed 10 Sept 2016).
13. Cole TJ, Freeman JV, Preece MA. British 1990 growth reference centiles for weight, height, body mass index and head circumference fitted by maximum penalized likelihood. StatMed. 1998;17(4):407-29.
